# Supplementary material for: Maternal n-3 enriched diet reprograms the offspring neurovascular transcriptome and blunts inflammation induced by endotoxin in the neonate
Source: J Neuroinflammation. 2024 Aug 11;21:199. doi: 10.1186/s12974-024-03191-8 (PMC11316986; doi:10.1186/s12974-024-03191-8)
Supplement: Supplementary file 1 — Supplementary Material 1 [file 12974_2024_3191_MOESM1_ESM.docx]

**Supplemental figures**

**
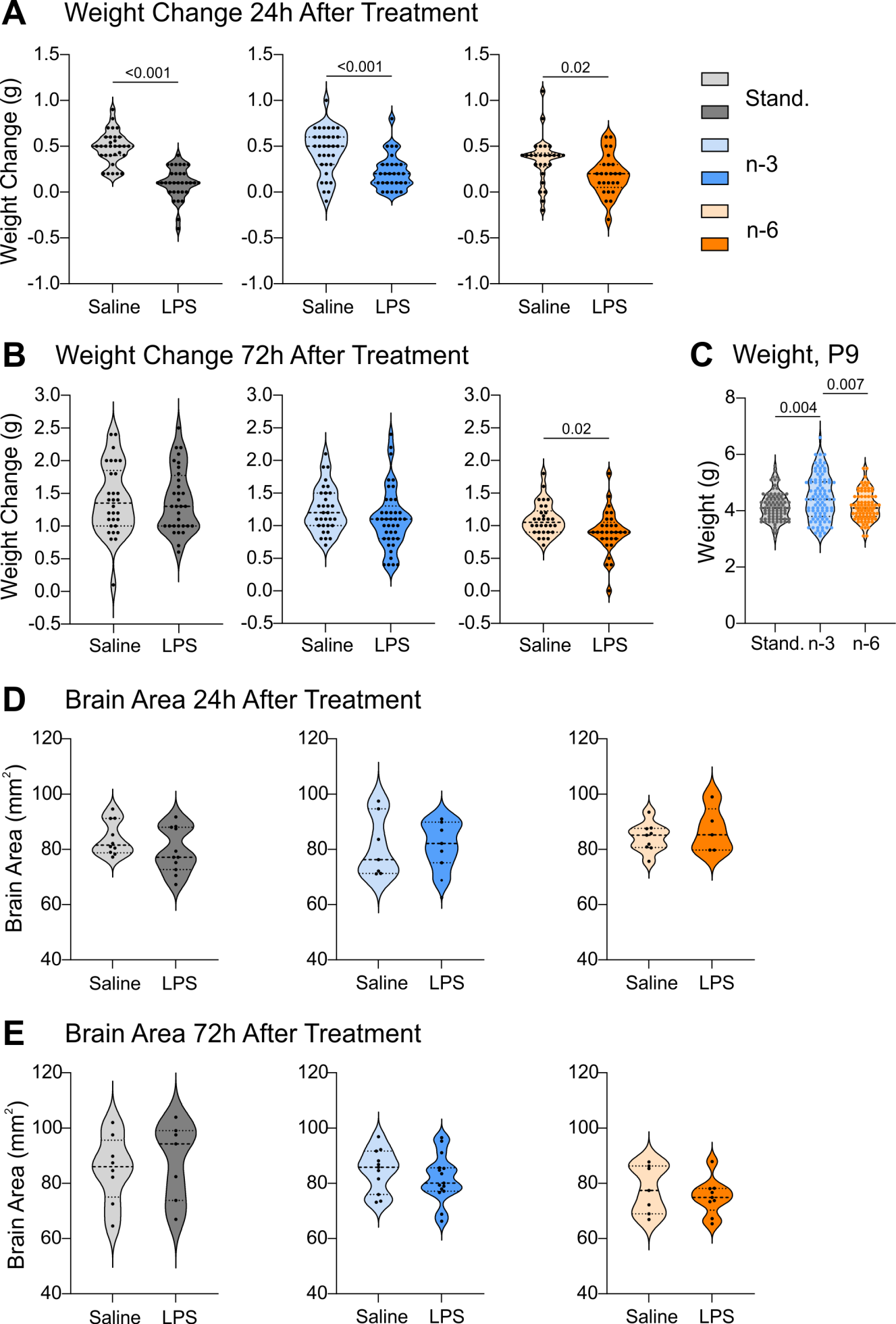
**

***Suppl. Figure 1****. N-6 diet affects post-LPS weight gain but not brain area in the offspring.* *Effect of systemic LPS administration on the weight gain in pups fed different diets. (A) Weight gain 24h after LPS or saline injection in pups from dams fed different diets, t-test. (B) Weight gain 72h after LPS or saline injection in pups from dams fed different diets, t-test. (C) Weight of pups from dams fed different diets at P9, at the time of LPS or saline injection. Welch's ANOVA, Dunnett's post-hoc test. (D) Axial brain area 24 h after injection. (E) Axial brain area 72 h after injection.*

*
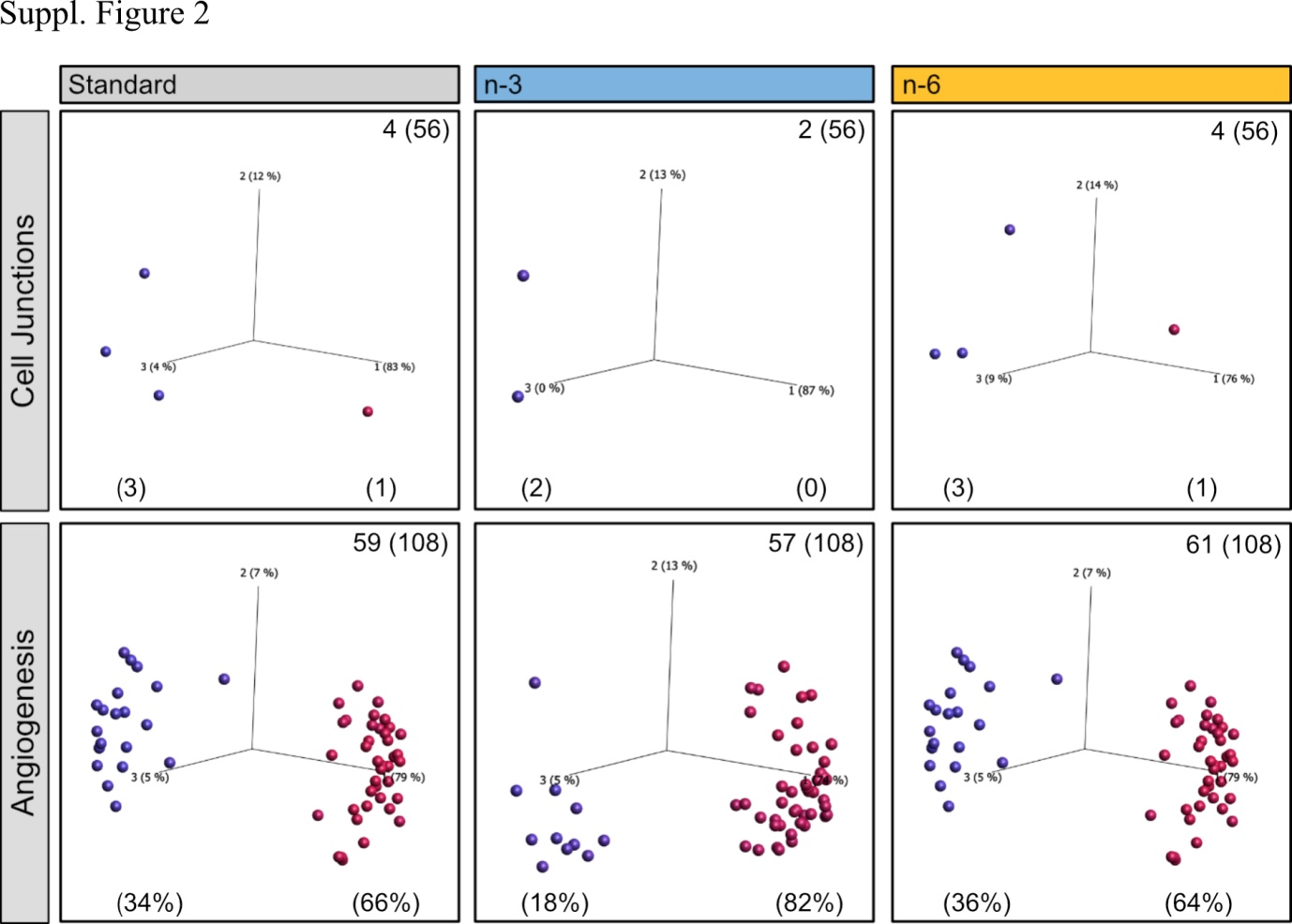
*

***Suppl. Figure 2****. Gene expression analysis suggests intact blood-brain barrier in all diets and a stronger pro-angiogenic response to LPS in n-3-supplemented diet group. PCA plots of individual genes annotated to cell junction and angiogenesis regulated by LPS within each diet. Variables are colour coded according to distance between them rendering upregulated genes red and downregulated genes blue. Numbers of regulated genes are stated in upper right corner out of all analysed genes for each process (in brackets).*

**
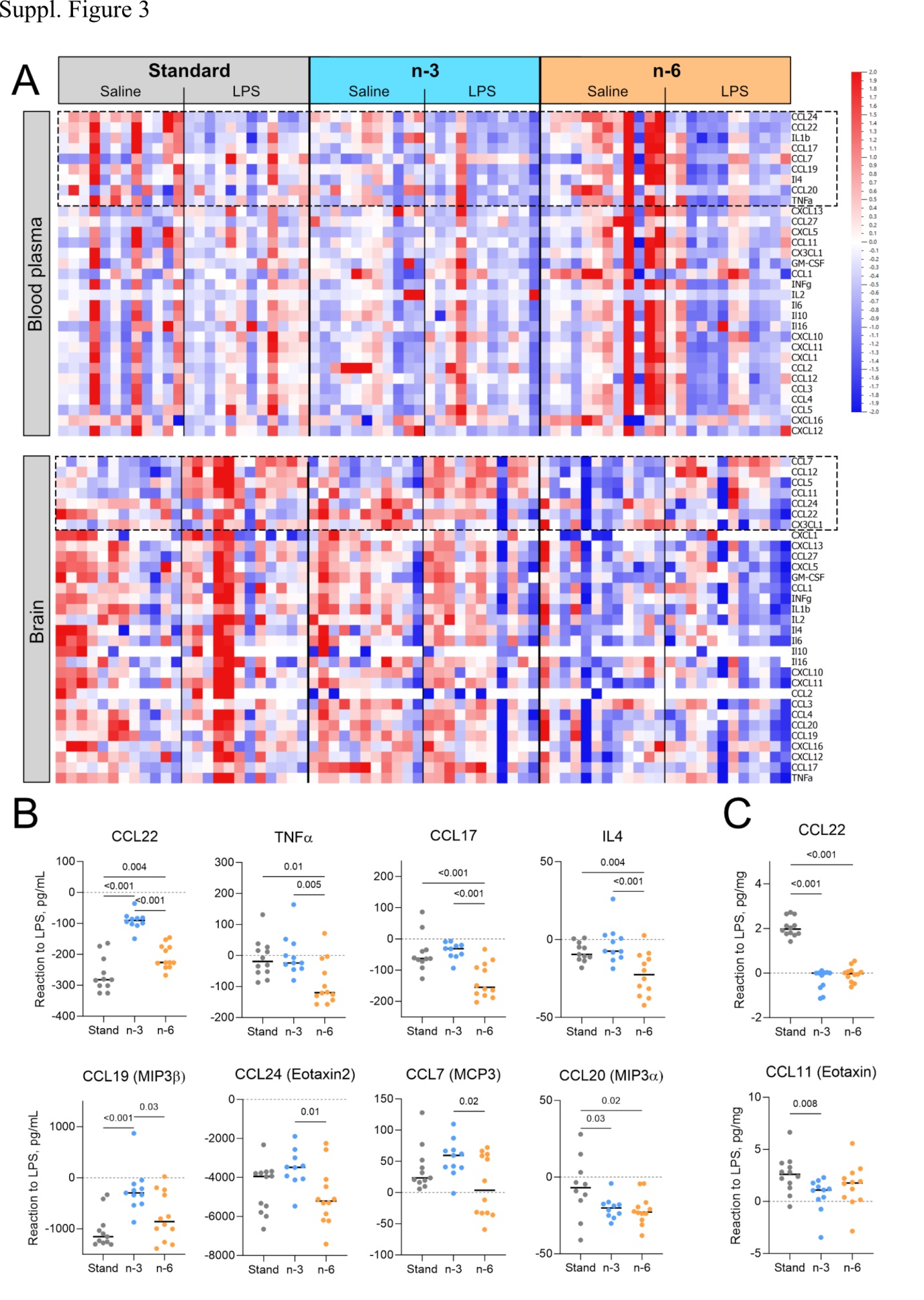
*Suppl. Figure 3****. PUFA diets modulate cytokine levels 72 h after LPS injection.* *Cytokine levels in the offspring blood plasma and brain 72 h after LPS injection. (A) Heatmap of cytokine levels analysed in blood plasma and brain of offspring from dams fed the standard, the n-3 or the n-6 enriched diet and injected with saline or LPS at P9. Each column refers to one animal and each row shows levels of a specific cytokine. Each cytokine level was normalized over all samples using Z-score normalization and is shown using a colour scheme based on Z-score distribution from − 2 to 2. Cytokines regulated by LPS (based on two-way ANOVA results) are highlighted with the frame. (B, C) Graphs showing the reaction to LPS in plasma (B) or brain (C). Zero level indicates no difference between the level of the cytokine in LPS sample and the mean level of the cytokine among the saline samples. Only graphs with significant differences between diet groups are presented. P values presented on the graphs show results of the post-hoc test. One-way ANOVA with Fisher’s LSD (B:IL4, CCL19, CCL24, CCL7, CCL20; C: CCL22) or Kruskal-Wallis with uncorrected Dunn’s (B: CCL22, TNFα, CCL17; C: CCL11 ) post hoc tests.*

**Supplemental Tables**

**Supplemental Table 1.** **Number of animals in each experimental group.**

| **Group** | **Males** | **Females** | **Total** |
| --- | --- | --- | --- |
| ***Gene expression data, samples (3 brains per sample)*** | | | |
| Standard saline | 1 | 3 | 4 |
| Standard LPS | 2 | 2 | 4 |
| N-3 saline | 3 | 1 | 4 |
| N-3 LPS | 3 | 2 | 5 |
| N-6 saline | 2 | 2 | 4 |
| N-6 LPS | 2 | 2 | 4 |
| ***Cytokine levels data, animals**** | | | |
| ***24h*** | | | |
| Standard saline | 6 | 6 | 12 |
| Standard LPS | 6 | 8 | 14 |
| N-3 saline | 6 | 6 | 12 |
| N-3 LPS | 6 | 6 | 12 |
| N-6 saline | 6 | 6 | 12 |
| N-6 LPS | 6 | 6 | 12 |
| ***72h*** | | | |
| Standard saline | 6 | 6 | 12 |
| Standard LPS | 6 | 6 | 12 |
| N-3 saline | 6 | 5 | 11 |
| N-3 LPS | 6 | 5 | 11 |
| N-6 saline | 6 | 6 | 12 |
| N-6 LPS | 6 | 6 | 12 |
| *Data for all animals were presented in the dataset. Outlier values for single cytokines were removed using the ROUT method. Outlier values were biologically implausible, likely reflecting technical issues. | | | |
| ***Vessel painting data, animals**** | | | |
| ***24h*** | | | |
| Standard saline | 6 | 4 | 10 |
| Standard LPS | 3 | 8 | 11 |
| N-3 saline | 5 | 2 | 7 |
| N-3 LPS | 6 | 1 | 7 |
| N-6 saline | 6 | 3 | 9 |
| N-6 LPS | 4 | 2 | 6 |
| ***72h*** | | | |
| Standard saline | 6 | 2 | 8 |
| Standard LPS | 5 | 2 | 7 |
| N-3 saline | 5 | 6 | 11 |
| N-3 LPS | 9 | 6 | 15 |
| N-6 saline | 4 | 3 | 7 |
| N-6 LPS | 5 | 4 | 9 |
|  |  |  |  |
| * Outliers removed using IQR test: For total vessel length: 1 male removed from standard saline 24h and 2 males from N-6 LPS 24h. For vessel density, 1 male was removed from N-3 LPS 72h. For branching density, 1 male was removed from N-3 LPS 72h. For average vessel length, 2 males were removed from N-3 LPS 72h and 1 male was removed from N-6 LPS 72h. For skewness and kurtosis, 1 male was removed from standard LPS 72h and 1 male from N-6 LPS 24h. | | | |

| **Supplemental Table 2.** Z-scores for selected top LPS regulated canonical pathways and biological functions in standard diet with comparisons to other diets generated by IPA. | | | | | | |
| --- | --- | --- | --- | --- | --- | --- |
|  |  |  |  |  |  |  |
|  | **Stand. diet** | | **n-3 diet** | | **n-6 diet** | |
| **Canonical pathway (c.p.)/biological function (b.f.)** | **z-score** | **Overrepresentation p value** | **z-score** | **Overrepresentation p value** | **z-score** | **Overrepresentation p value** |
| **Immune response** | | | | | | |
| Pathogen induced cytokine storm (c.p.) | + 3.18 | 6.31E-19 | + 5.74 | 6.31E-23 | + 5.11 | 3.98E-21 |
| Neuroinflammation (c.p.) | + 3.40 | 1.48E-09 | + 5.00 | 2.51E-18 | + 4.85 | 6.31E-15 |
| Antigen presentation (c.p.) | NA | 1.26E-13 | NA | 3.16E-14 | NA | 1.26E-17 |
| Granulocyte adhesion and diapedesis (c.p.) | NA | 1.26E-14 | NA | 1.26E-15 | NA | 1.26E-16 |
| Agranulocyte adhesion and diapedesis (c.p.) | NA | 1.12E-09 | NA | 1.76E-10 | NA | 3.16E-11 |
| Neutrophil extracellular trap (c.p.) | + 5.38 | 1.26E-11 | + 4.43 | 7.95E-13 | + 4.44 | 5.01E-12 |
| Macrophage classical activation (c.p.) | + 3.71 | 1.48E-07 | + 4.08 | 2.29E-10 | + 3.92 | 1.00E-12 |
| Cell death of immune cells (b.f.) | + 4.02 | 1.13E-16 | + 3.78 | 1.96E-24 | + 3.52 | 5.77E-22 |
| Cell movement of blood cells (b.f.) | + 3.52 | 2.13E-19 | + 4.57 | 7.60E-41 | + 4.64 | 2.31E-29 |
| Migration of blood cells (b.f.) | + 3.62 | 5.53E-19 | + 4.46 | 2.36E-40 | + 4.52 | 7.40E-29 |
| Adhesion of blood cells (b.f.) | + 4.53 | 1.33E-18 | + 5.81 | 4.27E-33 | + 5.70 | 7.45E-28 |
| Binding of blood cells (b.f.) | + 4.26 | 2.02E-18 | + 6.16 | 2.41E-32 | + 5.87 | 1.33E-26 |
| Viral life cycle (b.f.) | -3.53 | 4.22E-10 | -3.65 | 1.05E-12 | -3.09 | 2.43E-08 |
| Replication of virus (b.f.) | -1.88 | 2.32E-19 | -1.54 | 1.39E-21 | -0.45 | 4.03E-19 |
| **Cell death and survival** | | | | | | |
| Pyroptosis (c.p.) | + 3.30 | 1.95E-10 | + 4.26 | 3.98E-15 | + 4.58 | 1.00E-14 |
| Necrosis (b.f.) | + 4.77 | 1.8E-27 | + 3.11 | 2.66E-20 | + 2.39 | 4.71E-20 |
| Apoptosis of tumor cell lines (b.f.) | + 3.46 | 4.99E-19 | + 1.15 | 1.47E-14 | NA | 1.16E-11 |
| Cell survival (b.f.) | -2.58 | 2.53E-15 | + 1.20 | 2.11E-09 | + 2.22 | 8.29E-10 |
| Cell viability (b.f.) | -1.84 | 2.77E-13 | + 1.74 | 6.06E-08 | + 2.93 | 1.56E-08 |
| **Cell division and proliferation** | | | | | | |
| Cell proliferation of tumor cell lines (b.f.) | -3.48 | 1.87E-17 | + 2.12 | 5.44E-07 | + 2.507 | 1.14E-06 |
| Development of tumor cell lines (b.f.) | -1.76 | 1.08E-06 | NO | NO | NO | NO |
| M phase (b.f.) | -2.42 | 3.99E-12 | NO | NO | NO | NO |
| Alignment of chromosomes (b.f.) | -2.55 | 4.21E-13 | NO | NO | NO | NO |
| Repair of DNA (b.f.) | -3.24 | 4.8E-06 | NO | NO | NO | NO |
| Cytokinesis (b.f.) | -2.95 | 5.49E-08 | NO | NO | NO | NO |
| Cell cycle progression of tumor cell lines (b.f.) | -2.12 | 8.71E-07 | + 0.08 | 2.71E-05 | NO | NO |
| **Angiogenesis** | | | | | | |
| Angiogenesis (b.f.) | + 0.45 | 1.41E-09 | + 2.25 | 1.18E-07 | + 1.70 | 2.37E-08 |
|  |  |  |  |  |  |  |
| NA: no activity pattern available |  |  |  |  |  |  |
| NO: not overrepresented |  |  |  |  |  |  |

| **Supplemental Table 3.** Pathways enriched by LPS across diets generated by REACTOME |  | |
| --- | --- | --- |
|  |  | |
| **Standard diet** |  | |
| **Pathway name** | **Entities pValue** | |
| Interferon alpha/beta signaling | 1.110E-16 | |
| Interferon Signaling | 1.110E-16 | |
| Cytokine Signaling in Immune system | 1.110E-16 | |
| Immune System | 1.110E-16 | |
| Interferon gamma signaling | 3.664E-15 | |
| Interleukin-10 signaling | 1.242E-11 | |
| Signaling by Interleukins | 3.592E-9 | |
| Cell Cycle, Mitotic | 2.306E-7 | |
| Amplification of signal from the kinetochores | 6.311E-7 | |
| Amplification of signal from unattached kinetochores via a MAD2 inhibitory signal | 6.311E-7 | |
| Resolution of Sister Chromatid Cohesion | 1.447E-6 | |
| Neutrophil degranulation | 2.086E-6 | |
| Mitotic Spindle Checkpoint | 2.364E-6 | |
| Integrin cell surface interactions | 2.488E-6 | |
| Adaptive Immune System | 3.200E-6 | |
| Chemokine receptors bind chemokines | 3.655E-6 | |
| Cell Cycle Checkpoints | 1.461E-5 | |
| Polo-like kinase mediated events | 1.601E-5 | |
| Separation of Sister Chromatids | 1.633E-5 | |
| Mitotic Anaphase | 1.805E-5 | |
| Mitotic Metaphase and Anaphase | 1.954E-5 | |
| Mitotic Prometaphase | 2.675E-5 | |
| Interleukin-4 and Interleukin-13 signaling | 2.675E-5 | |
| EML4 and NUDC in mitotic spindle formation | 2.772E-5 | |
| Cell Cycle | 3.919E-5 | |
| Extracellular matrix organization | 4.239E-5 | |
| Antigen processing-Cross presentation | 1.704E-4 | |
| RHO GTPases Activate Formins | 1.812E-4 | |
| Immunoregulatory interactions between a Lymphoid and a non-Lymphoid cell | 2.097E-4 | |
| Pyroptosis | 2.998E-4 | |
| M Phase | 3.839E-4 | |
| ISG15 antiviral mechanism | 8.381E-4 | |
| Activation of C3 and C5 | 9.789E-4 | |
| Collagen degradation | 0.001 | |
| Mitotic G1 phase and G1/S transition | 0.001 | |
| CLEC7A/inflammasome pathway | 0.002 | |
| Collagen chain trimerization | 0.002 | |
| ER-Phagosome pathway | 0.003 | |
| Kinesins | 0.004 | |
| ECM proteoglycans | 0.004 | |
| Peptide ligand-binding receptors | 0.004 | |
| Regulation of mitotic cell cycle | 0.005 | |
| APC/C-mediated degradation of cell cycle proteins | 0.005 | |
| Degradation of the extracellular matrix | 0.005 | |
| MHC class II antigen presentation | 0.005 | |
| Class I MHC mediated antigen processing & presentation | 0.005 | |
| Signaling by ALK | 0.006 | |
| MyD88 deficiency (TLR2/4) | 0.006 | |
| Alternative complement activation | 0.006 | |
| RHO GTPase Effectors | 0.007 | |
| IRAK4 deficiency (TLR2/4) | 0.007 | |
| Regulation of TLR by endogenous ligand | 0.007 | |
| Regulated Necrosis | 0.008 | |
| Collagen biosynthesis and modifying enzymes | 0.008 | |
| Regulation of IFNA/IFNB signaling | 0.008 | |
| Negative regulators of DDX58/IFIH1 signaling | 0.008 | |
| Metal sequestration by antimicrobial proteins | 0.009 | |
| Apoptosis induced DNA fragmentation | 0.009 | |
| Assembly of collagen fibrils and other multimeric structures | 0.009 | |
| Interleukin-36 pathway | 0.009 | |
| Innate Immune System | 0.010 | |
| TP53 Regulates Transcription of Genes Involved in G2 Cell Cycle Arrest | 0.010 | |
| Interleukin-1 family signaling | 0.010 | |
| Collagen formation | 0.013 | |
| Condensation of Prometaphase Chromosomes | 0.014 | |
| MET activates PTK2 signaling | 0.015 | |
| Orc1 removal from chromatin | 0.016 | |
| Diseases of Immune System | 0.016 | |
| Diseases associated with the TLR signaling cascade | 0.016 | |
| DAP12 interactions | 0.016 | |
| COPI-dependent Golgi-to-ER retrograde traffic | 0.016 | |
| Platelet Adhesion to exposed collagen | 0.017 | |
| OAS antiviral response | 0.017 | |
| GPVI-mediated activation cascade | 0.018 | |
| Defective factor VIII causes hemophilia A | 0.018 | |
| TP53 Regulates Transcription of Cell Cycle Genes | 0.020 | |
| APC/C:Cdc20 mediated degradation of mitotic proteins | 0.020 | |
| Interleukin-6 signaling | 0.021 | |
| SARS-CoV-2 activates/modulates innate and adaptive immune responses | 0.021 | |
| Activation of APC/C and APC/C:Cdc20 mediated degradation of mitotic proteins | 0.022 | |
| Antiviral mechanism by IFN-stimulated genes | 0.022 | |
| G2/M Transition | 0.023 | |
| Programmed Cell Death | 0.023 | |
| Mitotic G2-G2/M phases | 0.025 | |
| Inactivation of CSF3 (G-CSF) signaling | 0.026 | |
| Interleukin-2 family signaling | 0.027 | |
| Molecules associated with elastic fibres | 0.028 | |
| RUNX1 regulates transcription of genes involved in differentiation of keratinocytes | 0.031 | |
| G0 and Early G1 | 0.031 | |
| Switching of origins to a post-replicative state | 0.032 | |
| SARS-CoV-1 activates/modulates innate immune responses | 0.033 | |
| Regulation of APC/C activators between G1/S and early anaphase | 0.034 | |
| Syndecan interactions | 0.034 | |
| Interleukin-20 family signaling | 0.034 | |
| Transcription of E2F targets under negative control by p107 (RBL1) and p130 (RBL2) in complex with HDAC1 | 0.035 | |
| Non-integrin membrane-ECM interactions | 0.036 | |
| GP1b-IX-V activation signalling | 0.038 | |
| Unwinding of DNA | 0.038 | |
| Interleukin-1 processing | 0.038 | |
| Regulation of ornithine decarboxylase (ODC) | 0.039 | |
| APC:Cdc20 mediated degradation of cell cycle proteins prior to satisfation of the cell cycle checkpoint | 0.040 | |
| TRAF3-dependent IRF activation pathway | 0.041 | |
| Diseases of hemostasis | 0.041 | |
| Defects of contact activation system (CAS) and kallikrein/kinin system (KKS) | 0.041 | |
| G1/S Transition | 0.045 | |
| Establishment of Sister Chromatid Cohesion | 0.046 | |
| Cross-presentation of particulate exogenous antigens (phagosomes) | 0.046 | |
| Cyclin A/B1/B2 associated events during G2/M transition | 0.048 | |
| **N-3 diet** |  |  |
| **Pathway name** | **Entities pValue** |  |
| Interferon alpha/beta signaling | 1.110E-16 |  |
| Interferon Signaling | 1.110E-16 |  |
| Innate Immune System | 1.110E-16 |  |
| Immune System | 1.110E-16 |  |
| Interferon gamma signaling | 1.110E-16 |  |
| Cytokine Signaling in Immune system | 1.110E-16 |  |
| Signaling by Interleukins | 1.110E-16 |  |
| Neutrophil degranulation | 3.330E-16 |  |
| Interleukin-10 signaling | 3.109E-15 |  |
| Interleukin-4 and Interleukin-13 signaling | 2.134E-9 |  |
| Chemokine receptors bind chemokines | 7.954E-8 |  |
| Adaptive Immune System | 2.403E-7 |  |
| Immunoregulatory interactions between a Lymphoid and a non-Lymphoid cell | 1.062E-6 |  |
| Interleukin-1 family signaling | 5.914E-6 |  |
| Trafficking and processing of endosomal TLR | 5.969E-6 |  |
| Signal regulatory protein family interactions | 1.384E-5 |  |
| DAP12 interactions | 1.662E-5 |  |
| Antigen processing-Cross presentation | 3.144E-5 |  |
| Activation of C3 and C5 | 6.582E-5 |  |
| Toll-like Receptor Cascades | 8.356E-5 |  |
| Insulin-like Growth Factor-2 mRNA Binding Proteins (IGF2BPs/IMPs/VICKZs) bind RNA | 1.345E-4 |  |
| SARS-CoV-1 activates/modulates innate immune responses | 2.707E-4 |  |
| Interleukin-37 signaling | 3.220E-4 |  |
| Peptide ligand-binding receptors | 4.954E-4 |  |
| Interleukin-2 family signaling | 5.353E-4 |  |
| CLEC7A/inflammasome pathway | 0.001 |  |
| ISG15 antiviral mechanism | 0.002 |  |
| Negative regulators of DDX58/IFIH1 signaling | 0.002 |  |
| The AIM2 inflammasome | 0.002 |  |
| RHO GTPases Activate NADPH Oxidases | 0.002 |  |
| Binding of TCF/LEF:CTNNB1 to target gene promoters | 0.003 |  |
| Interleukin-3, Interleukin-5 and GM-CSF signaling | 0.003 |  |
| SARS-CoV-2 activates/modulates innate and adaptive immune responses | 0.003 |  |
| PD-1 signaling | 0.004 |  |
| Phosphorylation of CD3 and TCR zeta chains | 0.004 |  |
| Pyroptosis | 0.004 |  |
| ER-Phagosome pathway | 0.004 |  |
| Purinergic signaling in leishmaniasis infection | 0.005 |  |
| Cell recruitment (pro-inflammatory response) | 0.005 |  |
| Alternative complement activation | 0.005 |  |
| DAP12 signaling | 0.006 |  |
| UNC93B1 deficiency - HSE | 0.006 |  |
| Generation of second messenger molecules | 0.007 |  |
| Cross-presentation of particulate exogenous antigens (phagosomes) | 0.007 |  |
| TRAF3-dependent IRF activation pathway | 0.008 |  |
| Toll Like Receptor 4 (TLR4) Cascade | 0.009 |  |
| Diseases of Immune System | 0.013 |  |
| Diseases associated with the TLR signaling cascade | 0.013 |  |
| Class A/1 (Rhodopsin-like receptors) | 0.013 |  |
| Integrin cell surface interactions | 0.013 |  |
| GPVI-mediated activation cascade | 0.014 |  |
| Signaling by ALK | 0.014 |  |
| TCR signaling | 0.014 |  |
| Antiviral mechanism by IFN-stimulated genes | 0.015 |  |
| OAS antiviral response | 0.015 |  |
| Interleukin-1 signaling | 0.017 |  |
| Cytosolic sensors of pathogen-associated DNA | 0.018 |  |
| MyD88 deficiency (TLR2/4) | 0.019 |  |
| Regulation of TLR by endogenous ligand | 0.020 |  |
| STAT3 nuclear events downstream of ALK signaling | 0.022 |  |
| IRAK4 deficiency (TLR2/4) | 0.022 |  |
| Transcriptional regulation of granulopoiesis | 0.023 |  |
| Platelet activation, signaling and aggregation | 0.025 |  |
| Regulation of IFNA/IFNB signaling | 0.025 |  |
| Interleukin-18 signaling | 0.027 |  |
| Interleukin receptor SHC signaling | 0.029 |  |
| Cell surface interactions at the vascular wall | 0.030 |  |
| Activation of IRF3, IRF7 mediated by TBK1, IKBKE | 0.031 |  |
| Translocation of ZAP-70 to Immunological synapse | 0.032 |  |
| Costimulation by the CD28 family | 0.034 |  |
| Interleukin-1 processing | 0.034 |  |
| Interleukin-21 signaling | 0.034 |  |
| Regulation of Complement cascade | 0.036 |  |
| Regulated Necrosis | 0.036 |  |
| Cross-presentation of soluble exogenous antigens (endosomes) | 0.038 |  |
| Binding and entry of HIV virion | 0.041 |  |
| Metal sequestration by antimicrobial proteins | 0.041 |  |
| TNFR2 non-canonical NF-kB pathway | 0.044 |  |
| DDX58/IFIH1-mediated induction of interferon-alpha/beta | 0.047 |  |
| Toll Like Receptor 7/8 (TLR7/8) Cascade | 0.047 |  |
| SARS-CoV-1-host interactions | 0.047 |  |
| Interleukin-38 signaling | 0.048 |  |
| FLT3 signaling through SRC family kinases | 0.048 |  |
| TICAM1-dependent activation of IRF3/IRF7 | 0.049 |  |
| NF-kB activation through FADD/RIP-1 pathway mediated by caspase-8 and − 10 | 0.049 |  |
| Interleukin-2 signaling | 0.049 |  |
| **N-6 diet** |  |  |
| **Pathway name** | **Entities pValue** |  |
| Interferon alpha/beta signaling | 1.110E-16 |  |
| Interferon Signaling | 1.110E-16 |  |
| Interferon gamma signaling | 1.110E-16 |  |
| Immune System | 1.110E-16 |  |
| Cytokine Signaling in Immune system | 1.110E-16 |  |
| Neutrophil degranulation | 4.885E-15 |  |
| Signaling by Interleukins | 1.288E-14 |  |
| Interleukin-10 signaling | 1.799E-14 |  |
| Innate Immune System | 1.932E-14 |  |
| Interleukin-4 and Interleukin-13 signaling | 2.995E-9 |  |
| Chemokine receptors bind chemokines | 8.054E-7 |  |
| Antigen processing-Cross presentation | 8.842E-7 |  |
| Adaptive Immune System | 2.129E-6 |  |
| Insulin-like Growth Factor-2 mRNA Binding Proteins (IGF2BPs/IMPs/VICKZs) bind RNA | 8.641E-6 |  |
| Trafficking and processing of endosomal TLR | 3.219E-5 |  |
| Activation of C3 and C5 | 4.566E-5 |  |
| ER-Phagosome pathway | 8.982E-5 |  |
| Integrin cell surface interactions | 9.328E-5 |  |
| Immunoregulatory interactions between a Lymphoid and a non-Lymphoid cell | 1.262E-4 |  |
| Toll-like Receptor Cascades | 3.528E-4 |  |
| Diseases associated with the TLR signaling cascade | 5.521E-4 |  |
| Diseases of Immune System | 5.521E-4 |  |
| MyD88 deficiency (TLR2/4) | 6.084E-4 |  |
| IRAK4 deficiency (TLR2/4) | 7.569E-4 |  |
| Peptide ligand-binding receptors | 8.684E-4 |  |
| CLEC7A/inflammasome pathway | 0.001 |  |
| Interleukin-1 family signaling | 0.001 |  |
| Transcriptional regulation of granulopoiesis | 0.001 |  |
| RUNX3 Regulates Immune Response and Cell Migration | 0.002 |  |
| DAP12 interactions | 0.002 |  |
| ISG15 antiviral mechanism | 0.002 |  |
| Pyroptosis | 0.003 |  |
| Purinergic signaling in leishmaniasis infection | 0.003 |  |
| Cell recruitment (pro-inflammatory response) | 0.003 |  |
| Signal regulatory protein family interactions | 0.003 |  |
| Regulation of TLR by endogenous ligand | 0.004 |  |
| Alternative complement activation | 0.004 |  |
| Regulation of IFNA/IFNB signaling | 0.005 |  |
| UNC93B1 deficiency - HSE | 0.005 |  |
| PD-1 signaling | 0.010 |  |
| The NLRP3 inflammasome | 0.010 |  |
| Inflammasomes | 0.011 |  |
| OAS antiviral response | 0.012 |  |
| TNFR2 non-canonical NF-kB pathway | 0.012 |  |
| Extracellular matrix organization | 0.013 |  |
| Interleukin-37 signaling | 0.014 |  |
| Generation of second messenger molecules | 0.015 |  |
| Negative regulators of DDX58/IFIH1 signaling | 0.016 |  |
| Activation of gene expression by SREBF (SREBP) | 0.016 |  |
| Inactivation of CSF3 (G-CSF) signaling | 0.016 |  |
| TCR signaling | 0.017 |  |
| STAT3 nuclear events downstream of ALK signaling | 0.017 |  |
| Class I MHC mediated antigen processing & presentation | 0.017 |  |
| SARS-CoV-1 activates/modulates innate immune responses | 0.017 |  |
| RHO GTPases Activate NADPH Oxidases | 0.018 |  |
| Class A/1 (Rhodopsin-like receptors) | 0.018 |  |
| Toll Like Receptor 4 (TLR4) Cascade | 0.019 |  |
| The AIM2 inflammasome | 0.020 |  |
| Regulation of Complement cascade | 0.021 |  |
| Regulated Necrosis | 0.024 |  |
| TNFs bind their physiological receptors | 0.024 |  |
| Antigen Presentation: Folding, assembly and peptide loading of class I MHC | 0.025 |  |
| Cross-presentation of soluble exogenous antigens (endosomes) | 0.026 |  |
| Interleukin-1 processing | 0.028 |  |
| Antiviral mechanism by IFN-stimulated genes | 0.028 |  |
| SARS-CoV-1-host interactions | 0.029 |  |
| SARS-CoV-2 activates/modulates innate and adaptive immune responses | 0.029 |  |
| GPVI-mediated activation cascade | 0.030 |  |
| Metal sequestration by antimicrobial proteins | 0.034 |  |
| Cross-presentation of particulate exogenous antigens (phagosomes) | 0.034 |  |
| Phosphorylation of CD3 and TCR zeta chains | 0.034 |  |
| Programmed Cell Death | 0.036 |  |
| Nucleotide-like (purinergic) receptors | 0.037 |  |
| ATF4 activates genes in response to endoplasmic reticulum stress | 0.038 |  |
| Potential therapeutics for SARS | 0.042 |  |
| Interleukin-38 signaling | 0.042 |  |
| Regulation of HMOX1 expression and activity | 0.042 |  |
| Signaling by CSF3 (G-CSF) | 0.042 |  |
| Interleukin-2 family signaling | 0.044 |  |
| Complement cascade | 0.046 |  |
| Regulation of cholesterol biosynthesis by SREBP (SREBF) | 0.049 |  |

| **Supplemental Table 4.** Descriptive statistics and results of two-way ANOVA analysis for cytokine levels in blood plasma of pups 24 h after LPS injection. | | | | | | | | | | | | | | | | | |
| --- | --- | --- | --- | --- | --- | --- | --- | --- | --- | --- | --- | --- | --- | --- | --- | --- | --- |
|  |  |  |  |  |  |  |  |  |  |  |  | | |  | |  | |
| **Plasma 24h** | **Standard** | | **n-3** | | **n-6** | | **Main effect of LPS treatment** | | **Main effect of diet** | | | **Post-hoc test p values, post-LPS levels** | | | | | |
| **Cytokine** | **Mean ± SD (pg/ml)** | | **Mean ± SD (pg/ml)** | | **Mean ± SD (pg/ml)** | |  |  |  |  |  |  |  |  |  |  |  |
|  | **Sal.** | **LPS** | **Sal.** | **LPS** | **Sal.** | **LPS** | **F (DFn. DFd)** | **p value** | **F (DFn. DFd)** | **p value** | **Stand. vs. n-3** | | | **Stand. vs. n-6** | | **n-3 vs. n-6** | |
| CXCL13 (BCA-1) | 674 ± 244.7 | 7401 ± 1882 | 818.9 ± 314.6 | 5740 ± 1984 | 739.8 ± 219.9 | 7557 ± 1355 | F (1. 63) = 362.9 | **< 0.001** | F (2. 63) = 2.72 | 0.074 | - | | | - | | - | |
| CCL27 | 3487 ± 1762 | 4708 ± 2250 | 3653 ± 1146 | 5140 ± 2818 | 5773 ± 3072 | 6414 ± 3384 | F (1. 69) = 2.65 | 0.108 | F (2. 69) = 5.05 | **0.009** | 0.235 | | | 0.468 | | 0.909 | |
| CXCL5 | 3013 ± 2760 | 1313 ± 1101 | 3020 ± 2871 | 1168 ± 1111 | 699.2 ± 191.8 | 524.6 ± 111.3 | F (1. 60) = 9.78 | **0.003** | F (2. 60) = 6.18 | **0.004** | 0.565 | | | 0.708 | | 0.978 | |
| CCL11 (Eotaxin) | 329.6 ± 152.8 | 383.9 ± 79.45 | 320.1 ± 56.82 | 477.6 ± 161.8 | 322.5 ± 113.4 | 424.9 ± 114.6 | F (1. 66) = 14.14 | **< 0.001** | F (2. 66) = 0.67 | 0.517 | - | | | - | | - | |
| CCL24 (Eotaxin2) | 3891 ± 1065 | 2146 ± 1019 | 3884 ± 1723 | 2362 ± 806.6 | 3763 ± 985.2 | 1720 ± 593.2 | F (1. 68) = 48.90 | **< 0.001** | F (2. 68) = 0.79 | 0.457 | - | | | - | | - | |
| CX3CL1 (Fraktalkine) | 348.1 ± 65.17 | 357.3 ± 36.86 | 342.4 ± 56.75 | 337.3 ± 77.83 | 341.6 ± 58.56 | 368.8 ± 38.80 | F (1. 67) = 0.71 | 0.401 | F (2. 67) = 0.74 | 0.48 | - | | | - | | - | |
| GM-CSF | 3.65 ± 2.32 | 2.797 ± 1.362 | 4.183 ± 2.000 | 3.298 ± 2.076 | 4.014 ± 1.568 | 4.019 ± 1.448 | F (1. 68) = 1.86 | 0.178 | F (2. 68) = 1.24 | 0.297 | - | | | - | | - | |
| CCL1 | 64.23 ± 20.78 | 76.78 ± 23.03 | 50.38 ± 10.15 | 62.88 ± 23.84 | 59.28 ± 13.87 | 72.48 ± 19.66 | F (1. 68) = 8.03 | **0.006** | F (2. 68) = 3.3 | **0.043** | 0.839 | | | 0.447 | | 0.168 | |
| INFγ | 149.8 ± 84.95 | 92.54 ± 42.88 | 150.7 ± 71.72 | 97.79 ± 72.50 | 124.9 ± 49.92 | 95.77 ± 40.53 | F (1. 68) = 10.07 | **0.002** | F (2. 68) = 0.03 | 0.973 | - | | | - | | - | |
| IL1β | 296.9 ± 110.4 | 221.9 ± 67.28 | 325.5 ± 90.66 | 242.4 ± 78.43 | 276.2 ± 56.87 | 220.0 ± 44.86 | F (1. 67) = 15.44 | **< 0.001** | F (2. 67) = 1.30 | 0.277 | - | | | - | | - | |
| IL2 | 11.79 ± 6.34 | 12.17 ± 5.930 | 17.07 ± 10.23 | 12.05 ± 6.825 | 11.35 ± 4.050 | 11.47 ± 3.133 | F (1. 64) = 0.99 | 0.324 | F (2. 64) = 1.51 | 0.229 | - | | | - | | - | |
| Il4 | 54.79 ± 15.82 | 44.65 ± 11.78 | 54.20 ± 16.83 | 43.72 ± 14.67 | 53.83 ± 13.66 | 48.97 ± 7.095 | F (1. 68) = 7.16 | **0.009** | F (2. 68) = 0.20 | 0.817 | - | | | - | | - | |
| Il6 | 84.75 ± 46.87 | 55.79 ± 24.20 | 80.72 ± 41.85 | 55.75 ± 38.19 | 63.99 ± 25.95 | 55.52 ± 22.68 | F (1. 67) = 6.69 | **0.012** | F (2. 67) = 0.65 | 0.527 | - | | | - | | - | |
| Il10 | 749.3 ± 402.6 | 735.6 ± 253.3 | 859.5 ± 431.8 | 642.3 ± 339.9 | 553.5 ± 247.1 | 714.8 ± 292.9 | F (1. 59) = 0.08 | 0.777 | F (2. 59) = 0.86 | 0.429 | - | | | - | | - | |
| Il16 | 722.9 ± 243.9 | 617.1 ± 142.7 | 756.8 ± 201.8 | 728.9 ± 306.1 | 675.2 ± 137.3 | 648.6 ± 138.2 | F (1. 66) = 1.26 | 0.266 | F (2. 66) = 1.12 | 0.334 | - | | | - | | - | |
| CXCL10 | 826.2 ± 277.2 | 7280 ± 2826 | 842.9 ± 223.8 | 7237 ± 5517 | 781.1 ± 127.2 | 6417 ± 2271 | F (1. 66) = 90.17 | **< 0.001** | F (2. 66) = 0.21 | 0.813 | - | | | - | | - | |
| CXCL11 | 665.3 ± 242.8 | 512.3 ± 156.9 | 655.5 ± 243.6 | 478.3 ± 254.1 | 574.3 ± 177.9 | 522.9 ± 150.8 | F (1. 68) = 6.7 | **0.012** | F (2. 68) = 0.39 | 0.68 | - | | | - | | - | |
| CXCL1 | 173.2 ± 87.70 | 114.9 ± 40.90 | 166.0 ± 80.88 | 153.5 ± 141.0 | 135.8 ± 46.02 | 114.8 ± 45.42 | F (1. 68) = 2.65 | 0.108 | F (2. 68) = 1.09 | 0.34 | - | | | - | | - | |
| CCL2 (MCP1) | 368.2 ± 150.2 | 590.4 ± 131.0 | 399.0 ± 164.8 | 695.2 ± 283.8 | 301.0 ± 76.23 | 587.3 ± 202.6 | F (1. 66) = 40.75 | **< 0.001** | F (2. 66) = 2 | 0.144 | - | | | - | | - | |
| CCL7 (MCP3) | 143.0 ± 62.23 | 4645 ± 1938 | 140.3 ± 38.07 | 4920 ± 3225 | 134.7 ± 39.27 | 3586 ± 1286 | F (1. 66) = 117.1 | **< 0.001** | F (2. 66) = 1.06 | 0.354 | - | | | - | | - | |
| CCL12 | 30.61 ± 10.00 | 218.7 ± 64.33 | 33.55 ± 7.316 | 258.6 ± 116.4 | 32.08 ± 7.960 | 242.9 ± 81.88 | F (1. 66) = 183.0 | **< 0.001** | F (2. 66) = 0.67 | 0.514 | - | | | - | | - | |
| CCL22 | 226.0 ± 102.1 | 221.4 ± 48.36 | 194.6 ± 75.04 | 262.5 ± 121.7 | 230.9 ± 73.90 | 254.8 ± 56.95 | F (1. 67) = 3.24 | 0.077 | F (2. 67) = 0.73 | 0.486 | - | | | - | | - | |
| CCL3 (MIP1α) | 13.68 ± 8.17 | 14.52 ± 4.05 | 13.41 ± 7.241 | 16.58 ± 12.55 | 10.36 ± 5.090 | 11.83 ± 4.73 | F (1. 67) = 2.05 | 0.157 | F (2. 67) = 1.15 | 0.323 | - | | | - | | - | |
| CCL4 (MIP1 | 119.7 ± 44.67 | 137.0 ± 33.73 | 127.3 ± 45.44 | 145.6 ± 54.35 | 109.7 ± 33.74 | 131.7 ± 39.92 | F (1. 67) = 3.77 | **0.057** | F (2. 67) = 0.82 | 0.446 | - | | | - | | - | |
| CCL20 (MIP3 | 78.94 ± 30.36 | 100.5 ± 38.75 | 65.23 ± 17.02 | 101.9 ± 41.24 | 86.26 ± 26.30 | 123.2 ± 35.34 | F (1. 67) = 16.82 | **0.001** | F (2. 67) = 2.59 | 0.082 | - | | | - | | - | |
| CCL19 (MIP3 | 1439 ± 425.8 | 1346 ± 264.4 | 1494 ± 451.6 | 1341 ± 454.1 | 1415 ± 377.7 | 1302 ± 252.5 | F (1. 68) = 1.85 | 0.179 | F (2. 68) = 0.15 | 0.862 | - | | | - | | - | |
| CCL5 (RANTES) | 57.02 ± 29.41 | 856.6 ± 264.6 | 58.64 ± 17.34 | 876.3 ± 454.3 | 52.82 ± 11.55 | 847.7 ± 229.7 | F (1. 66) = 202.2 | **< 0.001** | F (2. 66) = 0.03 | 0.97 | - | | | - | | - | |
| CXCL16 | 534.2 ± 222.6 | 833.4 ± 276.0 | 577.1 ± 188.1 | 887.9 ± 346.5 | 668.0 ± 211.0 | 955.0 ± 186.6 | F (1. 68) = 27.23 | **< 0.001** | F (2. 68) = 1.7 | 0.191 | - | | | - | | - | |
| CXCL12 | 722.8 ± 153.8 | 688.9 ± 173.6 | 946.1 ± 271.5 | 867.1 ± 275.6 | 797.9 ± 240.7 | 870.8 ± 145.8 | F (1. 62) = 0.02 | 0.901 | F (2. 62) = 4.63 | **0.013** | **0.03** | | | 0.792 | | 0.17 | |
| CCL17 | 136.8 ± 72.46 | 67.12 ± 17.73 | 147.1 ± 73.24 | 89.86 ± 61.86 | 120.8 ± 43.94 | 76.77 ± 35.91 | F (1. 67) = 28.49 | **< 0.001** | F (2. 67) = 0.44 | 0.644 | - | | | - | | - | |
| TNFa | 124.8 ± 66.69 | 83.46 ± 23.83 | 127.9 ± 62.89 | 67.91 ± 27.59 | 98.54 ± 35.38 | 94.79 ± 29.10 | F (1. 64) = 7.33 | **0.009** | F (2. 64) = 0.43 | 0.65 | - | | | - | | - | |
|  |  |  |  |  |  |  |  |  |  |  |  | | |  | |  | |
| P values lower than 0.05 are highlighted in bold | | | |  |  |  |  |  |  |  |  | | |  | |  | |
| Post-hoc analysis was performed for LPS-treated groups only for cytokines, for which the main effect of diet on the cytokine level was found to be significant using two-way ANOVA analysis | | | | | | | | | | | | |  | |  | |  |

| **Supplemental Table 5.** Descriptive statistics and results of two-way ANOVA analysis for cytokine levels in blood plasma of pups 72 h after LPS injection. | | | | | | | | | | | | | | | | |  |  |  |  | |
| --- | --- | --- | --- | --- | --- | --- | --- | --- | --- | --- | --- | --- | --- | --- | --- | --- | --- | --- | --- | --- | --- |
|  |  |  |  |  |  | |  |  | |  |  |  |  |  |  | |  |  |  |  |  |
| **Plasma 72h** | **Standard** | | **n-3** | | **n-6** | | | | **Main effect of LPS treatment** | | **Main effect of diet** | | **Post-hoc test p values, post-LPS levels** | | | |  |  |  |  |  |
| **Cytokine** | **Mean ± SD (pg/ml)** | | **Mean ± SD (pg/ml)** | | **Mean ± SD (pg/ml)** | | | |  |  |  |  |  |  |  |  |  |  |  |  |  |
|  | **Sal.** | **LPS** | **Sal.** | **LPS** | **Sal.** | | **LPS** | **F (DFn. DFd)** | | **p value** | **F (DFn. DFd)** | **p value** | **Stand. vs. n-3** | **Stand. vs. n-6** | **n-3 vs. n-6** | |  |  |  |  |  |
| CXCL13 (BCA-1) | 1024 ± 264.5 | 1091 ± 265.4 | 1044 ± 209.9 | 900.1 ± 128.1 | 1125 ± 404.8 | | 988.2 ± 264.1 | F (1. 62) = 1.15 | | 0.288 | F (2. 62) = 0.68 | 0.511 | - | - | - | |  |  |  |  |  |
| CCL27 | 4822 ± 1164 | 5073 ± 1610 | 5604 ± 1832 | 4930 ± 1853 | 5800 ± 1617 | | 5027 ± 1354 | F (1. 62) = 1.08 | | 0.304 | F (2. 62) = 0.52 | 0.595 | - | - | - | |  |  |  |  |  |
| CXCL5 | 2396 ± 2890 | 2279 ± 1665 | 798.6 ± 655.5 | 1066 ± 622.3 | 3005 ± 2413 | | 1417 ± 1349 | F (1. 62) = 0.12 | | 0.732 | F (2. 62) = 3.53 | **0.035** | 0.284 | 0.203 | 0.986 | |  |  |  |  |  |
| CCL11 (Eotaxin) | 329.9 ± 121.0 | 349.9 ± 80.66 | 340.1 ± 54.51 | 325.1 ± 27.24 | 390.2 ± 185.6 | | 320.9 ± 99.63 | F (1. 61) = 0.08 | | 0.785 | F (2. 61) = 0.01 | 0.99 | - | - | - | |  |  |  |  |  |
| CCL24 (Eotaxin2) | 9878 ± 2845 | 5347 ± 1271 | 8220 ± 2058 | 4681 ± 971.7 | 10265 ± 4165 | | 5272 ± 1485 | F (1. 63) = 55.01 | | **< 0.001** | F (2. 63) = 1.92 | 0.155 | - | - | - | |  |  |  |  |  |
| CX3CL1 (Fraktalkine) | 196.9 ± 52.53 | 179.7 ± 19.60 | 181.6 ± 21.82 | 176.1 ± 28.67 | 209.9 ± 53.08 | | 176.9 ± 29.00 | F (1. 62) = 3.67 | | 0.06 | F (2. 62) = 0.71 | 0.494 | - | - | - | |  |  |  |  |  |
| GM-CSF | 3.291 ± 2.379 | 3.3 ± 1.255 | 2.602 ± 2.08 | 2.773 ± 2.25 | 3.807 ± 3.987 | | 2.492 ± 2.11 | F (1. 64) = 0.40 | | 0.528 | F (2. 64) = 0.37 | 0.693 | - | - | - | |  |  |  |  |  |
| CCL1 | 46.27 ± 9.38 | 44.54 ± 7.62 | 50.52 ± 15.87 | 52.91 ± 10.49 | 59.84 ± 22.59 | | 50.82 ± 15.04 | F (1. 64) = 0.65 | | 0.423 | F (2. 64) = 2.91 | 0.062 | - | - | - | |  |  |  |  |  |
| INFγ | 288.2 ± 156.2 | 287.5 ± 94.00 | 218.7 ± 86.32 | 247.8 ± 127.1 | 404.5 ± 274.4 | | 223.9 ± 128.7 | F (1. 64) = 1.2 | | 0.278 | F (2. 64) = 1.13 | 0.331 | - | - | - | |  |  |  |  |  |
| IL1β | 325.4 ± 87.86 | 245.0 ± 53.25 | 315.5 ± 76.08 | 249.1 ± 68.11 | 349.3 ± 132.2 | | 243.8 ± 83.17 | F (1. 63) = 15.79 | | **< 0.001** | F (2. 63) = 0.17 | 0.843 | - | - | - | |  |  |  |  |  |
| IL2 | 38.84 ± 13.46 | 31.83 ± 19.35 | 37.87 ± 12.20 | 34.23 ± 11.72 | 35.33 ± 18.56 | | 22.87 ± 15.71 | F (1. 60) = 3.91 | | 0.053 | F (2. 60) = 1.34 | 0.271 | - | - | - | |  |  |  |  |  |
| Il4 | 48.29 ± 17.01 | 40.43 ± 6.209 | 39.60 ± 10.96 | 36.44 ± 11.96 | 60.07 ± 33.69 | | 37.79 ± 13.73 | F (1. 62) = 6.36 | | **0.014** | F (2. 62) = 1.76 | 0.181 | - | - | - | |  |  |  |  |  |
| Il6 | 82.08 ± 48.21 | 77.94 ± 27.83 | 60.23 ± 25.89 | 65.95 ± 36.79 | 111.5 ± 83.85 | | 58.06 ± 36.81 | F (1. 64) = 1.69 | | 0.199 | F (2. 64) = 1.05 | 0.356 | - | - | - | |  |  |  |  |  |
| Il10 | 644.9 ± 331.1 | 611.4 ± 171.3 | 522.6 ± 206.8 | 488.9 ± 270.2 | 836.0 ± 531.4 | | 452.4 ± 256.3 | F (1. 64) = 3.87 | | 0.053 | F (2. 64) = 1.28 | 0.286 | - | - | - | |  |  |  |  |  |
| Il16 | 648.6 ± 309.0 | 563.5 ± 129.3 | 540.7 ± 73.60 | 529.3 ± 115.4 | 560.7 ± 123.0 | | 557.7 ± 218.2 | F (1. 61) = 0.59 | | 0.446 | F (2. 61) = 0.55 | 0.58 | - | - | - | |  |  |  |  |  |
| CXCL10 | 603.4 ± 160.7 | 705.1 ± 153.8 | 579.7 ± 101.9 | 709.8 ± 161.5 | 752.1 ± 309.1 | | 605.1 ± 190.6 | F (1. 64) = 0.77 | | 0.382 | F (2. 64) = 0.02 | 0.98 | - | - | - | |  |  |  |  |  |
| CXCL11 | 3271 ± 1413 | 3363 ± 1170 | 2704 ± 936.7 | 2876 ± 995.9 | 3838 ± 1438 | | 2592 ± 1100 | F (1. 63) = 1.3 | | 0.26 | F (2. 63) = 1.24 | 0.296 | - | - | - | |  |  |  |  |  |
| CXCL1 | 210.5 ± 110.4 | 207.6 ± 63.74 | 162.9 ± 56.77 | 180.3 ± 63.15 | 236.8 ± 124.3 | | 166.7 ± 72.75 | F (1. 63) = 0.34 | | 0.56 | F (2. 63) = 1.03 | 0.362 | - | - | - | |  |  |  |  |  |
| CCL2 (MCP1) | 465.9 ± 222.8 | 457.5 ± 157.8 | 753.4 ± 631.0 | 545.0 ± 333.1 | 525.5 ± 252.0 | | 343.1 ± 167.4 | F (1. 63) = 2.34 | | 0.131 | F (2. 63) = 1.08 | 0.346 | - | - | - | |  |  |  |  |  |
| CCL7 (MCP3) | 152.4 ± 56.05 | 178.7 ± 20.40 | 130.8 ± 24.96 | 185.9 ± 29.51 | 167.5 ± 90.86 | | 178.1 ± 49.39 | F (1. 62) = 10.22 | | **0.002** | F (2. 62) = 0.12 | 0.891 | - | - | - | |  |  |  |  |  |
| CCL12 | 36.62 ± 10.30 | 35.80 ± 6.38 | 35.29 ± 4.11 | 36.65 ± 7.53 | 39.79 ± 18.57 | | 33.05 ± 9 | F (1. 64) = 0.68 | | 0.413 | F (2. 64) = 0.011 | 0.989 | - | - | - | |  |  |  |  |  |
| CCL22 | 261.5 ± 193.0 | 175.7 ± 63.26 | 227.3 ± 60.66 | 135.7 ± 28.39 | 273.6 ± 207.9 | | 133.4 ± 38.90 | F (1. 62) = 56.51 | | **< 0.001** | F (2. 62) = 2.33 | 0.106 | - | - | - | |  |  |  |  |  |
| CCL3 (MIP1α) | 17.31 ± 11.34 | 17.20 ± 5.44 | 12.59 ± 5.94 | 14.41 ± 6.53 | 25.16 ± 19.90 | | 12.22 ± 7.48 | F (1. 64) = 0.77 | | 0.385 | F (2. 64) = 0.81 | 0.45 | - | - | - | |  |  |  |  |  |
| CCL4 (MIP1 | 99.53 ± 45.88 | 93.52 ± 28.71 | 79.90 ± 21.79 | 77.40 ± 28.21 | 108.9 ± 53.07 | | 71.79 ± 30.44 | F (1. 63) = 3.01 | | 0.088 | F (2. 63) = 1.42 | 0.25 | - | - | - | |  |  |  |  |  |
| CCL20 (MIP3 | 63.39 ± 28.27 | 57.54 ± 19.59 | 66.91 ± 21.19 | 46.75 ± 7.2 | 76.69 ± 37.69 | | 55.38 ± 9.74 | F (1. 63) = 5.26 | | **0.025** | F (2. 63) = 0.8 | 0.454 | - | - | - | |  |  |  |  |  |
| CCL19 (MIP3 | 1777 ± 511.1 | 1407 ± 384.5 | 1483 ± 292.2 | 1242 ± 432.8 | 1984 ± 942.0 | | 1220 ± 474.4 | F (1. 63) = 11.68 | | **0.001** | F (2. 63) = 1.26 | 0.29 | - | - | - | |  |  |  |  |  |
| CCL5 (RANTES) | 83.07 ± 23.23 | 97.56 ± 27.97 | 69.26 ± 12.44 | 96.54 ± 29.79 | 93.96 ± 27.87 | | 82.68 ± 23.87 | F (1. 62) = 2.99 | | 0.089 | F (2. 62) = 0.7 | 0.502 | - | - | - | |  |  |  |  |  |
| CXCL16 | 721.5 ± 82.42 | 634.8 ± 81.50 | 664.5 ± 125.6 | 678.2 ± 97.69 | 659.5 ± 256.4 | | 656.9 ± 70.62 | F (1. 63) = 0.59 | | 0.447 | F (2. 63) = 0.13 | 0.878 | - | - | - | |  |  |  |  |  |
| CXCL12 | 645.0 ± 189.1 | 721.0 ± 238.5 | 616.3 ± 248.8 | 492.7 ± 92.44 | 835.3 ± 478.0 | | 520.4 ± 177.3 | F (1. 60) = 2.77 | | 0.101 | F (2. 60) = 1.80 | 0.174 | - | - | - | |  |  |  |  |  |
| CCL17 | 182.3 ± 105.0 | 124.9 ± 45.37 | 121.5 ± 45.14 | 84.28 ± 26.32 | 233.4 ± 161.4 | | 100.7 ± 55.38 | F (1. 63) = 15.47 | | **< 0.001** | F (2. 63) = 3.34 | **0.042** | 0.178 | 0.32 | 0.91 | |  |  |  |  |  |
| TNFa | 148.6 ± 75.16 | 123.8 ± 39.45 | 103.2 ± 48.73 | 103.6 ± 65.97 | 178.0 ± 112.4 | | 90.49 ± 72.54 | F (1. 64) = 4.80 | | **0.032** | F (2. 64) = 1.56 | 0.218 | - | - | - | |  |  |  |  |  |
|  |  |  |  |  |  | |  |  | |  |  |  |  |  |  | |  |  |  |  |  |
| P values lower than 0.05 are highlighted in bold | | | | | | | | | | | | | | | | |  |  |  |  |  |
| Post-hoc analysis was performed for LPS-treated groups only for cytokines, for which the main effect of diet on the cytokine level was found to be significant using two-way ANOVA analysis | | | | | | | | | | | | | | | | |  |  |  | |  |
| **Supplemental Table 6.** Descriptive statistics and results of two-way ANOVA analysis for cytokine levels in brain of pups 24 h after LPS injection. | | | | | | | | | | | | | | | | |  |  |  |  | |
|  |  | |  |  |  |  |  |  | |  |  |  |  |  | |  |  |  |  |  |  |
| **Brain 24h** | **Standard** | | **n-3** | | **n-6** | | | | **Main effect of LPS treatment** | | **Main effect of diet** | | **Post-hoc test p values, post-LPS levels** | | | |  |  |  |  |  |
| **Cytokine** | **Mean ± SD (pg/mg)** | | **Mean ± SD (pg/mg)** | | **Mean ± SD (pg/mg)** | | | |  |  |  |  |  |  |  |  |  |  |  |  |  |
|  | **Sal.** | **LPS** | **Sal.** | **LPS** | **Sal.** | | **LPS** | **F (DFn. DFd)** | | **p value** | **F (DFn. DFd)** | **p value** | **Stand. vs. n-3** | **Stand. vs. n-6** | | **n-3 vs. n-6** |  |  |  |  |  |
| CXCL13 (BCA-1) | 159.1 ± 31.41 | 202.0 ± 47.54 | 119.9 ± 14.33 | 155.2 ± 43.11 | 123.9 ± 17.14 | | 173.5 ± 23.10 | F (1. 68) = 31.61 | | **< 0.001** | F (2. 68) = 11.82 | **< 0.001** | **0.001** | 0.073 | | 0.358 |  |  |  |  |  |
| CCL27 | 543.1 ± 111.2 | 532.0 ± 132.4 | 420.7 ± 65.93 | 391.3 ± 79.51 | 455.0 ± 80.05 | | 457.1 ± 82.08 | F (1. 68) = 0.33 | | 0.568 | F (2. 68) = 11.98 | **< 0.001** | **0.001** | 0.124 | | 0.22 |  |  |  |  |  |
| CXCL5 | 62.45 ± 12.56 | 62.86 ± 15.10 | 51.83 ± 10.96 | 45.59 ± 8.01 | 52.00 ± 11.33 | | 56.59 ± 7.87 | F (1. 68) = 0.02 | | 0.877 | F (2. 68) = 9.50 | **< 0.001** | **< 0.001** | 0.346 | | 0.054 |  |  |  |  |  |
| CCL11 (Eotaxin) | 9.19 ± 1.97 | 15.12 ± 4.14 | 7.95 ± 1.68 | 13.20 ± 3.29 | 7.45 ± 1.29 | | 13.23 ± 3.64 | F (1. 68) = 69.25 | | **< 0.001** | F (2. 68) = 2.88 | 0.063 | - | - | | - |  |  |  |  |  |
| CCL24 (Eotaxin2) | 1120 ± 237.0 | 1764 ± 527.1 | 782.8 ± 59.45 | 1193 ± 344.4 | 765.8 ± 86.28 | | 1381 ± 128.5 | F (1. 67) = 64.58 | | **< 0.001** | F (2. 67) = 16.59 | **< 0.001** | **< 0.001** | **0.005** | | 0.267 |  |  |  |  |  |
| CX3CL1 (Fraktalkine) | 374.2 ± 61.29 | 343.0 ± 80.67 | 315.0 ± 47.50 | 263.8 ± 43.71 | 329.4 ± 39.64 | | 320.5 ± 50.39 | F (1. 68) = 5.34 | | **0.024** | F (2. 68) = 9.33 | **< 0.001** | **0.002** | 0.571 | | **0.043** |  |  |  |  |  |
| GM-CSF | 0.25 ± 0.08 | 0.24 ± 0.07 | 0.2 ± 0.05 | 0.16 ± 0.04 | 0.2 ± 0.05 | | 0.19 ± 0.04 | F (1. 68) = 2.91 | | 0.093 | F (2. 68) = 10.01 | **< 0.001** | **0.001** | 0.06 | | 0.36 |  |  |  |  |  |
| CCL1 | 4.79 ± 0.94 | 4.57 ± 1.45 | 3.55 ± 0.54 | 3.45 ± 0.67 | 3.86 ± 0.89 | | 3.86 ± 0.91 | F (1. 68) = 0.22 | | 0.641 | F (2. 68) = 9.98 | **< 0.001** | **0.012** | 0.149 | | 0.559 |  |  |  |  |  |
| INFγ | 16.99 ± 3.5 | 19.27 ± 5.79 | 11.89 ± 1.87 | 13.69 ± 3.11 | 13.56 ± 3.30 | | 15.50 ± 3.32 | F (1. 68) = 5.29 | | **0.025** | F (2. 68) = 13.23 | **< 0.001** | **< 0.001** | **0.034** | | 0.469 |  |  |  |  |  |
| IL1β | 52.12 ± 10.37 | 66.41 ± 16.42 | 39.49 ± 5.43 | 43.67 ± 9.53 | 41.15 ± 9.09 | | 52.22 ± 9.17 | F (1. 68) = 15.49 | | **< 0.001** | F (2. 68) = 18.04 | **< 0.001** | **< 0.001** | **0.004** | | 0.132 |  |  |  |  |  |
| IL2 | 6.03 ± 1.89 | 6.51 ± 2.26 | 4.45 ± 0.95 | 3.79 ± 0.82 | 4.15 ± 1.28 | | 4.16 ± 0.74 | F (1. 68) = 0.03 | | 0.864 | F (2. 68) = 17.64 | **< 0.001** | **< 0.001** | **< 0.001** | | 0.815 |  |  |  |  |  |
| Il4 | 10.94 ± 1.86 | 10.52 ± 2.79 | 8.15 ± 1.18 | 7.46 ± 1.62 | 8.88 ± 2.11 | | 9.29 ± 2.06 | F (1. 68) = 0.25 | | 0.617 | F (2. 68) = 13.13 | **< 0.001** | **< 0.001** | 0.28 | | 0.075 |  |  |  |  |  |
| Il6 | 5.44 ± 1.13 | 5.74 ± 1.74 | 3.95 ± 0.65 | 3.92 ± 0.75 | 4.05 ± 1.1 | | 4.40 ± 0.87 | F (1. 68) = 0.63 | | 0.431 | F (2. 68) = 15.53 | **< 0.001** | **< 0.001** | **0.009** | | 0.555 |  |  |  |  |  |
| Il10 | 38.27 ± 19.85 | 42.23 ± 17.79 | 19.73 ± 8.39 | 25.80 ± 12.35 | 27.83 ± 15.17 | | 27.56 ± 14.95 | F (1. 67) = 0.56 | | 0.459 | F (2. 67) = 8.03 | **< 0.001** | **0.021** | **0.044** | | 0.956 |  |  |  |  |  |
| Il16 | 301.3 ± 51.55 | 301.3 ± 70.07 | 269.0 ± 64.35 | 218.2 ± 51.11 | 267.7 ± 56.54 | | 260.7 ± 53.76 | F (1. 68) = 2 | | 0.163 | F (2. 68) = 6.21 | **0.003** | **0.002** | 0.191 | | 0.187 |  |  |  |  |  |
| CXCL10 | 204.9 ± 34.08 | 233.8 ± 47.81 | 167.5 ± 25.77 | 210.7 ± 98.52 | 177.6 ± 28.51 | | 195.8 ± 22.50 | F (1. 68) = 6.63 | | **0.012** | F (2. 68) = 3.31 | **0.044** | 0.476 | 0.14 | | 0.747 |  |  |  |  |  |
| CXCL11 | 196.4 ± 23.71 | 202.8 ± 46.68 | 167.4 ± 48.46 | 131.8 ± 36.16 | 170.6 ± 40.77 | | 178.2 ± 22.15 | F (1. 68) = 0.66 | | 0.42 | F (2. 68) = 10.75 | **< 0.001** | **< 0.001** | 0.236 | | **0.011** |  |  |  |  |  |
| CXCL1 | 6.39 ± 1.63 | 6.72 ± 1.61 | 4.59 ± 1.09 | 5.03 ± 1.24 | 5.14 ± 1.28 | | 6.05 ± 0.86 | F (1. 68) = 3.35 | | 0.071 | F (2. 68) = 10.86 | **< 0.001** | **0.005** | 0.413 | | 0.149 |  |  |  |  |  |
| CCL2 (MCP1) | 29.83 ± 4.94 | 120.9 ± 22.26 | 16.33 ± 7.86 | 118.6 ± 59.49 | 21.01 ± 7.36 | | 140.6 ± 59.12 | F (1. 60) = 130.1 | | **< 0.001** | F (2. 60) = 0.61 | 0.548 | - | - | | - |  |  |  |  |  |
| CCL7 (MCP3) | 8.18 ± 1.37 | 102.1 ± 24.50 | 6.68 ± 1.17 | 103.7 ± 45.86 | 7.19 ± 0.9 | | 93.10 ± 10.08 | F (1. 67) = 322.7 | | **< 0.001** | F (2. 67) = 0.42 | 0.658 | - | - | | - |  |  |  |  |  |
| CCL12 | 3.87 ± 1.26 | 37.94 ± 11.92 | 3.01 ± 0.42 | 37.90 ± 27.00 | 3.83 ± 1.05 | | 34.49 ± 8.95 | F (1. 67) = 124.5 | | **< 0.001** | F (2. 67) = 0.13 | 0.882 | - | - | | - |  |  |  |  |  |
| CCL22 | 2.7 ± 0.50 | 5 ± 1.2 | 2.02 ± 0.32 | 3.97 ± 0.71 | 2.21 ± 0.44 | | 4.99 ± 0.9 | F (1. 66) = 168.5 | | **< 0.001** | F (2. 66) = 7.66 | **0.001** | **0.004** | > 0.999 | | **0.006** |  |  |  |  |  |
| CCL3 (MIP1α) | 5.94 ± 1.13 | 6.73 ± 1.86 | 5.35 ± 0.81 | 6.6 ± 1.76 | 5.99 ± 1.09 | | 7.72 ± 2.19 | F (1. 68) = 11.98 | | **0.001** | F (2. 68) = 1.95 | 0.15 | - | - | | - |  |  |  |  |  |
| CCL4 (MIP1 | 10.50 ± 2.37 | 13.23 ± 3.17 | 7.48 ± 1.69 | 8.55 ± 1.65 | 7.75 ± 2.57 | | 10.91 ± 0.92 | F (1. 67) = 19.65 | | **< 0.001** | F (2. 67) = 19.03 | **< 0.001** | **< 0.001** | **0.027** | | **0.031** |  |  |  |  |  |
| CCL20 (MIP3 | 6.73 ± 1.35 | 6.77 ± 1.85 | 5.26 ± 1.01 | 4.61 ± 0.68 | 5.22 ± 0.88 | | 5.18 ± 0.84 | F (1. 68) = 0.62 | | 0.433 | F (2. 68) = 16.94 | **< 0.001** | **< 0.001** | **0.004** | | 0.473 |  |  |  |  |  |
| CCL19 (MIP3 | 297.8 ± 54.99 | 527.2 ± 151.7 | 245.2 ± 28.61 | 381.7 ± 120.8 | 240.8 ± 38.61 | | 398.0 ± 55.23 | F (1. 68) = 69.09 | | **< 0.001** | F (2. 68) = 9.59 | **< 0.001** | **< 0.001** | **0.002** | | 0.898 |  |  |  |  |  |
| CCL5 (RANTES) | 3.83 ± 1.03 | 24.73 ± 6.60 | 2.42 ± 0.62 | 17.88 ± 6.99 | 2.64 ± 0.73 | | 24.11 ± 4.14 | F (1. 65) = 335.7 | | **< 0.001** | F (2. 65) = 5.44 | **0.007** | **< 0.001** | 0.933 | | **0.003** |  |  |  |  |  |
| CXCL16 | 8.72 ± 1.42 | 11.51 ± 2.01 | 7.08 ± 0.88 | 9.65 ± 2.97 | 7.23 ± 0.85 | | 10.25 ± 0.85 | F (1. 68) = 49.84 | | **< 0.001** | F (2. 68) = 7.45 | **0.001** | **0.019** | 0.148 | | 0.666 |  |  |  |  |  |
| CXCL12 | 175.8 ± 32.61 | 177.3 ± 44.69 | 140.6 ± 21.53 | 129.1 ± 20.72 | 151.5 ± 27.22 | | 150.9 ± 23.74 | F (1. 68) = 0.25 | | 0.616 | F (2. 68) = 12.16 | **< 0.001** | **< 0.001** | **0.074** | | 0.187 |  |  |  |  |  |
| CCL17 | 15.77 ± 3.08 | 15.98 ± 3.93 | 12.67 ± 2.3 | 10.82 ± 2.08 | 14.26 ± 2.22 | | 14.59 ± 3.09 | F (1. 67) = 0.41 | | 0.526 | F (2. 67) = 12.50 | **< 0.001** | **< 0.001** | 0.451 | | **0.008** |  |  |  |  |  |
| TNFa | 31.30 ± 5.94 | 33.3 ± 8.89 | 24.30 ± 3.01 | 24.73 ± 4.67 | 26.02 ± 5.37 | | 30.42 ± 4.57 | F (1. 68) = 2.82 | | 0.098 | F (2. 68) = 11.17 | **< 0.001** | **0.001** | 0.423 | | 0.05 |  |  |  |  |  |
|  |  | |  |  |  |  |  |  | |  |  |  |  |  | |  |  |  |  |  |  |
| P values lower than 0.05 are highlighted are highlighted in bold | | | | | | | | | | | | | | | | |  |  |  |  |  |
| Post-hoc analysis was performed for LPS-treated groups only for cytokines, for which the main effect of diet on the cytokine level was found to be significant using two-way ANOVA analysis | | | | | | | | | | | | | | | | |  |  |  | |  |
| **Supplemental Table 7.** Descriptive statistics and results of two-way ANOVA analysis for cytokine levels in brain of pups 72 h after LPS injection. | | | | | | | | | | | | | | | | |  |  |  |  | |
|  |  | |  |  |  |  |  |  | |  |  |  |  |  | |  |  |  |  |  |  |
| **Brain 72h** | **Standard** | | **n-3** | | **n-6** | | | | **Main effect of LPS treatment** | | **Main effect of diet** | | **Post-hoc test p values, post-LPS levels** | | | |  |  |  |  |  |
| **Cytokine** | **Mean ± SD (pg/mg)** | | **Mean ± SD (pg/mg)** | | **Mean ± SD (pg/mg)** | | | |  |  |  |  |  |  |  |  |  |  |  |  |  |
|  | **Sal.** | **LPS** | **Sal.** | **LPS** | **Sal.** | | **LPS** | **F (DFn. DFd)** | | **p value** | **F (DFn. DFd)** | **p value** | **Stand. vs. n-3** | **Stand. vs. n-6** | | **n-3 vs. n-6** |  |  |  |  |  |
| CXCL13 (BCA-1) | 86.27 ± 11.40 | 89.66 ± 10.73 | 87.28 ± 10.51 | 84.23 ± 15.66 | 82.23 ± 20.14 | | 71.81 ± 14.45 | F (1. 68) = 1 | | 0.32 | F (2. 68) = 4.07 | **0.021** | 0.617 | **0.008** | | 0.079 |  |  |  |  |  |
| CCL27 | 325.4 ± 64.65 | 328.2 ± 71.82 | 316.0 ± 49.36 | 315.3 ± 69.64 | 313.2 ± 76.13 | | 264.5 ± 66.91 | F (1. 68) = 0.98 | | 0.326 | F (2. 68) = 2.11 | 0.129 | - | - | | - |  |  |  |  |  |
| CXCL5 | 35.65 ± 10.12 | 32.78 ± 9.822 | 30.11 ± 9.07 | 30.13 ± 9.77 | 23.43 ± 8.1 | | 25.97 ± 14.34 | F (1. 67) = 0.002 | | 0.967 | F (2. 67) = 5.19 | **0.008** | 0.799 | 0.249 | | 0.578 |  |  |  |  |  |
| CCL11 (Eotaxin) | 8.07 ± 0.75 | 10.64 ± 1.808 | 9.15 ± 1.01 | 10.07 ± 1.7 | 7.85 ± 1.43 | | 9.17 ± 1.96 | F (1. 68) = 16.49 | | **< 0.001** | F (2. 68) = 3.87 | **0.026** | 0.693 | 0.069 | | 0.312 |  |  |  |  |  |
| CCL24 (Eotaxin2) | 1432 ± 116.8 | 1296 ± 184.3 | 1470 ± 244.5 | 1247 ± 121.0 | 1441 ± 256.4 | | 1209 ± 299.3 | F (1. 67) = 15.01 | | **< 0.001** | F (2. 67) = 0.24 | 0.787 | - | - | | - |  |  |  |  |  |
| CX3CL1 (Fraktalkine) | 443.5 ± 35.74 | 456.4 ± 61.23 | 471.1 ± 44.11 | 428.5 ± 25.42 | 463.5 ± 100.1 | | 430.5 ± 63.08 | F (1. 66) = 4.65 | | **0.035** | F (2. 66) = 0.13 | 0.877 | - | - | | - |  |  |  |  |  |
| GM-CSF | 0.06 ± 0.03 | 0.059 ± 0.024 | 0.05 ± 0.03 | 0.06 ± 0.02 | 0.04 ± 0.01 | | 0.04 ± 0.02 | F (1. 65) = 1.07 | | 0.304 | F (2. 65) = 5.40 | **0.007** | 0.853 | 0.18 | | 0.061 |  |  |  |  |  |
| CCL1 | 3.96 ± 0.54 | 4.272 ± 0.999 | 3.95 ± 0.60 | 3.9 ± 0.70 | 3.79 ± 0.86 | | 3.46 ± 0.98 | F (1. 68) = 0.02 | | 0.898 | F (2. 68) = 2.38 | 0.101 | - | - | | - |  |  |  |  |  |
| INFγ | 8.98 ± 1.38 | 9.320 ± 2.105 | 8.81 ± 1.37 | 8.30 ± 2.03 | 8.14 ± 1.95 | | 7.32 ± 1.31 | F (1. 67) = 0.65 | | 0.422 | F (2. 67) = 4.11 | **0.021** | 0.315 | **0.017** | | 0.342 |  |  |  |  |  |
| IL1β | 28.32 ± 7.54 | 26.63 ± 5.2 | 28.26 ± 6.29 | 25.12 ± 7.5 | 24.31 ± 8.64 | | 21.15 ± 6.56 | F (1. 67) = 2.58 | | 0.113 | F (2. 67) = 3.18 | **0.048** | 0.855 | 0.148 | | 0.347 |  |  |  |  |  |
| IL2 | 2.26 ± 0.91 | 2.875 ± 1.492 | 2.59 ± 0.90 | 2.64 ± 0.85 | 2.34 ± 1.33 | | 1.71 ± 0.79 | F (1. 67) = 0.19 | | 0.667 | F (2. 67) = 3.82 | **0.027** | 0.942 | **0.047** | | **0.015** |  |  |  |  |  |
| Il4 | 7.23 ± 2.70 | 7.497 ± 2.227 | 7.23 ± 2.2 | 6.99 ± 1.24 | 6.50 ± 1.83 | | 6.29 ± 1.29 | F (1. 67) = 0.08 | | 0.783 | F (2. 67) = 0.88 | 0.42 | - | - | | - |  |  |  |  |  |
| Il6 | 2.43 ± 0.75 | 2.459 ± 0.593 | 2.28 ± 0.78 | 2.30 ± 0.72 | 1.90 ± 0.51 | | 1.83 ± 0.63 | F (1. 67) = 0.01 | | 0.918 | F (2. 67) = 4.74 | **0.012** | 0.737 | **0.04** | | 0.178 |  |  |  |  |  |
| Il10* | 12.25 ± 4.65 | 15.46 ± 5.278 | 2.07 ± 1.19 | 3.86 ± 2.54 | / | | / | F (1. 13) = 0.01 | | 0.916 | F (2. 13) = 4.92 | **0.026** | - | - | | - |  |  |  |  |  |
| Il16 | 122.4 ± 37.05 | 145.1 ± 57.54 | 137.8 ± 41.62 | 134.8 ± 28.82 | 129.6 ± 45.15 | | 117.8 ± 38.78 | F (1. 68) = 0.04 | | 0.853 | F (2. 68) = 0.83 | 0.441 | - | - | | - |  |  |  |  |  |
| CXCL10 | 159.1 ± 16.10 | 162.9 ± 25.28 | 158.2 ± 15.70 | 148.5 ± 21.33 | 150.7 ± 28.34 | | 142.2 ± 22.35 | F (1. 68) = 0.91 | | 0.344 | F (2. 68) = 2.73 | 0.073 | - | - | | - |  |  |  |  |  |
| CXCL11 | 118.1 ± 35.86 | 121.7 ± 34.94 | 103.5 ± 32.78 | 115.2 ± 30.57 | 84.61 ± 28.86 | | 86.99 ± 28.10 | F (1. 66) = 0.62 | | 0.434 | F (2. 66) = 7.13 | **0.002** | 0.868 | **0.03** | | 0.086 |  |  |  |  |  |
| CXCL1* | 2.021 ± 1.05 | 1.602 ± 1.282 | 1.93 ± 0.41 | 2.12 ± 0.73 | 0.281 ± 0.04 | | 1.71 ± 0.92 | F (1. 39) = 0.91 | | 0.346 | F (2. 39) = 2.23 | 0.121 | - | - | | - |  |  |  |  |  |
| CCL2 (MCP1)* | 8.70 ± 2.81 | 12.42 ± 4.909 | 4.77 ± 3.00 | 3.79 ± 2.22 | / | | / | F (1. 17) = 0.17 | | 0.686 | F (2. 17) = 9.14 | **0.002** | - | - | | - |  |  |  |  |  |
| CCL7 (MCP3) | 8.25 ± 0.97 | 11.53 ± 1.528 | 8.04 ± 1.11 | 10.54 ± 1.03 | 7.86 ± 1.42 | | 10.81 ± 2.42 | F (1. 66) = 76.62 | | **< 0.001** | F (2. 66) = 2.39 | 0.1 | - | - | | - |  |  |  |  |  |
| CCL12 | 2.73 ± 0.9 | 4.501 ± 1.492 | 2.56 ± 0.48 | 4.36 ± 1.01 | 2.46 ± 0.81 | | 4.08 ± 1.26 | F (1. 68) = 44.43 | | **< 0.001** | F (2. 68) = 0.59 | 0.557 | - | - | | - |  |  |  |  |  |
| CCL22 | 2.2 ± 0.42 | 2.056 ± 0.422 | 2.42 ± 0.42 | 2.1 ± 0.43 | 1.91 ± 0.32 | | 1.79 ± 0.30 | F (1. 67) = 4.84 | | **0.031** | F (2. 67) = 7.14 | **0.002** | 0.966 | 0.213 | | 0.124 |  |  |  |  |  |
| CCL3 (MIP1α) | 10.09 ± 0.76 | 10.38 ± 0.788 | 10.88 ± 1.53 | 10.57 ± 0.93 | 10.99 ± 0.96 | | 9.68 ± 1.78 | F (1. 65) = 1.48 | | 0.228 | F (2. 65) = 0.97 | 0.384 | - | - | | - |  |  |  |  |  |
| CCL4 (MIP1 | 7.78 ± 1.48 | 7.945 ± 1.767 | 7.7 ± 1.14 | 7.71 ± 1.45 | 6.7 ± 1.74 | | 6.39 ± 1.12 | F (1. 66) = 0.02 | | 0.879 | F (2. 66) = 5.48 | **0.006** | 0.949 | 0.058 | | 0.114 |  |  |  |  |  |
| CCL20 (MIP3 | 4.93 ± 0.81 | 4.860 ± 0.848 | 4.70 ± 0.78 | 4.54 ± 1.12 | 4.39 ± 1.32 | | 3.84 ± 0.76 | F (1. 68) = 1.31 | | 0.257 | F (2. 68) = 4.18 | **0.019** | 0.686 | **0.029** | | 0.169 |  |  |  |  |  |
| CCL19 (MIP3 | 184.9 ± 26.09 | 177.8 ± 28.39 | 188.0 ± 22.37 | 170.3 ± 34.51 | 176.7 ± 43.45 | | 156.7 ± 25.79 | F (1. 68) = 3.65 | | 0.06 | F (2. 68) = 1.73 | 0.186 | - | - | | - |  |  |  |  |  |
| CCL5 (RANTES) | 3.69 ± 0.51 | 4.856 ± 0.751 | 3.48 ± 0.85 | 4.35 ± 0.82 | 3.30 ± 0.88 | | 3.74 ± 1.3 | F (1. 67) = 25.14 | | **< 0.001** | F (2. 67) = 3.77 | **0.028** | 0.242 | **0.029** | | 0.552 |  |  |  |  |  |
| CXCL16 | 10.65 ± 1.99 | 10.47 ± 1.656 | 10.39 ± 1.52 | 10.24 ± 0.54 | 9.78 ± 2.1 | | 9.71 ± 1.77 | F (1. 65) = 0.04 | | 0.834 | F (2. 65) = 1.07 | 0.35 | - | - | | - |  |  |  |  |  |
| CXCL12 | 105.1 ± 12.15 | 106.8 ± 17.14 | 105.0 ± 9.11 | 99.05 ± 14.70 | 98.77 ± 20.51 | | 97.05 ± 14.22 | F (1. 68) = 0.32 | | 0.574 | F (2. 68) = 1.89 | 0.159 | - | - | | - |  |  |  |  |  |
| CCL17 | 14.41 ± 1.95 | 14.73 ± 1.989 | 17.50 ± 3.80 | 16.41 ± 4.23 | 14.59 ± 2.22 | | 12.29 ± 3.69 | F (1. 68) = 1.98 | | 0.164 | F (2. 68) = 8.10 | **< 0.001** | 0.378 | 0.133 | | **0.004** |  |  |  |  |  |
| TNFa | 11.45 ± 2.03 | 11.96 ± 3.069 | 11.36 ± 1.50 | 10.78 ± 2.66 | 9.95 ± 2.14 | | 9.85 ± 2.97 | F (1. 67) = 0.01 | | 0.924 | F (2. 67) = 3.35 | **0.041** | 0.466 | 0.092 | | 0.602 |  |  |  |  |  |
|  |  | |  |  |  |  |  |  | |  |  |  |  |  | |  |  |  |  |  |  |
|  |  | |  |  |  |  |  |  | |  |  |  |  |  | |  |  |  |  |  |  |
| P values lower than 0.05 are highlighted in bold | | | | | | | | | | | | | | | | |  |  |  |  |  |
| Post-hoc analysis was performed for LPS-treated groups only for cytokines, for which the main effect of diet on the cytokine level was found to be significant using two-way ANOVA analysis | | | | | | | | | | | | | | | | |  |  |  | |  |
| *Multiple samples are missing in all groups due to levels not reaching the lower limit of quantification of the assay | | | | | | | | | | | | | | | | |  |  |  |  | |
